# Osimertinib-Induced Hepatitis Following Immunotherapy in a Patient with Lung Adenocarcinoma Harboring De Novo EGFR Exon 19 Deletion and T790M Mutations: A Case Report

**DOI:** 10.3390/reports8030101

**Published:** 2025-06-26

**Authors:** Bradley Steiner, Amanda Edmond, Monica Camou, Taylor Praska, Jiaxin Niu

**Affiliations:** 1 Department of Internal Medicine, University of Arizona College of Medicine Phoenix, Phoenix, AZ 85004, USA; bradleysteiner@arizona.edu; 2 Division of Medical Oncology, Banner M.D. Anderson Cancer Center, Gilbert, AZ 85234, USA; amanda.edmond@bannerhealth.com (A.E.); monica.camou3@bannerhealth.com (M.C.); taylor.praska@bannerhealth.com (T.P.)

**Keywords:** lung cancer, immunotherapy, EGFR, TKI, irAE, osimertinib, hepatitis

## Abstract

**Background and Clinical Significance:** Non-small-cell lung cancer (NSCLC) with EGFR mutations, particularly de novo compound mutations such as exon 19 deletions (Ex19del) with T790M substitutions, present a significant clinical challenge due to resistance to many treatments. While treating these patients, the administration of osimertinib, a third-generation EGFR inhibitor, after immunotherapy can lead to unique immune-related adverse events (irAEs), such as pneumonitis and, rarely, hepatitis. **Case Presentation:** A 36-year-old Filipino woman presented with metastatic NSCLC harboring de novo Ex19del and T790M mutations. Despite initial therapy with carboplatin and paclitaxel, followed by chemoimmunotherapy, the patient’s disease progressed. She subsequently developed severe hepatitis from osimertinib after her prior immunotherapy with pembrolizumab. After the hepatitis resolved with high-dose steroids, osimertinib was switched to afatinib, but her disease rapidly progressed with new metastases. A second attempt at osimertinib rechallenge, with concomitant prednisone, resulted in substantial disease control, including improved leptomeningeal disease (LMD) and no recurrence of hepatitis. **Conclusions:** This case underscores the feasibility of rechallenging with osimertinib in patients who experience adverse events such as hepatotoxicity, provided that appropriate management strategies, such as steroid therapy, are employed. The successful rechallenge in this case highlights the potential of osimertinib as a viable option in advanced EGFR-mutant NSCLC, even after prior treatment-related complications.

## 1. Introduction and Clinical Significance

Mutations in the epidermal growth factor receptor (*EGFR*) gene are found in approximately 10–15% of NSCLC cases in Western populations and 30–40% or more of NSCLC cases in Asian populations [1,2]. Common activating mutations, such as exon 19 deletions (Ex19del) and L858R substitutions, are typically sensitive to EGFR-specific tyrosine kinase inhibitors (TKIs). As a result, EGFR-TKIs, either as monotherapy or in combination, are well-established first-line treatment options in EGFR-mutant NSCLC [3,4].

Treatment becomes more complex when activating mutations co-exist with resistance mutations such as T790M or C797S; requiring more advanced therapeutic strategies to overcome resistance and improve patient outcomes [5]. However, combining or sequencing EGFR-TKIs with other treatment modalities such as immunotherapy (IO) can have unique and significant challenges. The combination of programmed cell death protein 1 (PD-1) inhibitors with EGFR-TKIs, for example, has been associated with severe immune related adverse events (irAEs) such as pneumonitis [6]. Understanding these risks, exploring how to best sequence various treatment modalities, and learning to manage adverse events when they do occur will be vital as the use of both IO and TKIs grows.

Here, we report a case of acute hepatitis following the administration of the third-generation EGFR-TKI osimertinib in a patient who had previously received IO with pembrolizumab for metastatic NSCLC. We discuss the challenges in treatment selection due to the patient’s compound de novo EGFR mutations, management of her osimertinib-induced hepatitis, and the successful re-introduction of osimertinib following the failure of alternative therapies.

## 2. Case Presentation

A 36-year-old Filipino woman with no smoking history presented to a local emergency department in May of 2022 following 8 weeks of chest pain and progressively worsening dyspnea. Imaging showed a large left-sided pleural effusion and subsequent workup revealed biopsy-proven lung adenocarcinoma with multiple brain metastases. Next-generation sequencing (NGS) identified compound de novo mutations in the *EGFR* gene: Ex19del (S752_I759 variant) and T790M. PD-L1 was positive with a tumor proportion score of 1%.

Initial treatment options were impacted by insurance concerns and her original care team opted for whole-brain radiation followed by combination chemotherapy with carboplatin and paclitaxel. However, due to recurrent left-sided pleural effusions, in August 2022 her systemic therapy was switched to a chemoimmunotherapy regimen combining carboplatin/pemetrexed alongside pembrolizumab. Follow-up imaging after four cycles of chemoimmunotherapy demonstrated a partial response and the patient was transitioned to maintenance pemetrexed and pembrolizumab.

**Figure 1:**
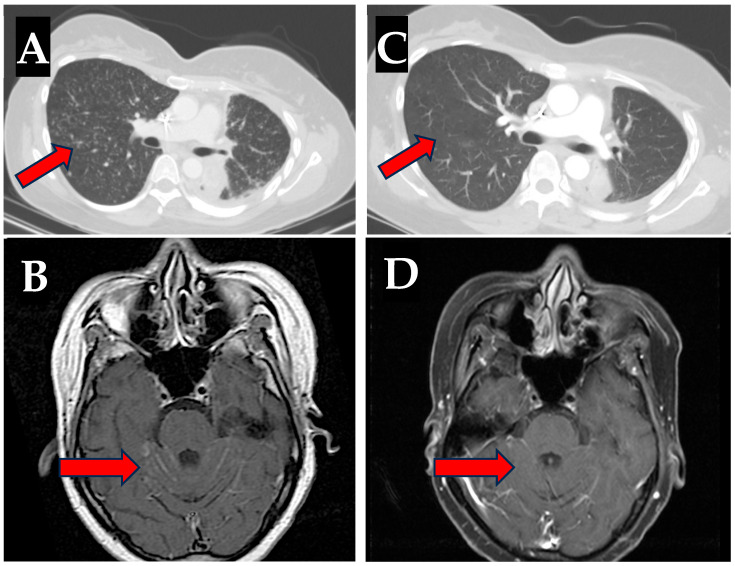
Rapid improvement to lymphangitic and leptomeningeal disease with osimertinib. Lymphangitic disease in the lung (**A**) and leptomeningeal disease burden (**B**) while on afatinib therapy (June 2023 and July 2023, respectively); leptomeningeal carcinomatosis was noted within the cerebral and cerebellar hemispheres on MRI and innumerable bilateral pulmonary nodules were appreciated on CT. Improvement to both lymphangitic lung metastases on CT (**C**) and to leptomeningeal disease (**D**) on MRI after osimertinib rechallenge (August 2023 and September 2023, respectively).

Following her last cycle of maintenance therapy on 16 December 2022, the patient developed severe left-sided chest wall pain. Imaging of the chest, abdomen, and pelvis demonstrated significant disease progression. Osimertinib 80 mg by mouth (PO) daily was subsequently started on 10 January 2023. The patient tolerated this well initially but she was admitted to the hospital on 22 February 2023 with fever, worsening fatigue, and right midback pain. Laboratory studies revealed markedly elevated AST (1113 U/L), ALT (411 U/L), alkaline phosphatase (1088 U/L), and bilirubinemia (3.7 mg/dL). The patient was a never drinker, with negative hepatitis A, B, and C serologies, baseline ALT and AST < 20 U/L on previous labs, and with no known history of previous liver injury. A liver biopsy was taken which demonstrated diffuse lymphocyte infiltration consistent with immune-mediated hepatitis. The patient responded well to high-dose steroids with normalization of liver function following discontinuation of osimertinib and a four-week steroid taper. Targeted therapy was then switched to afatinib in April of 2023, and although she did not experience further hepatotoxicity, her disease rapidly progressed with lymphangitic spread throughout the lungs and significant leptomeningeal disease burden (LMD) (Figure 1A,B), as well as metastases to the liver and bone.

In July 2023, the patient transferred her care to our facility. Liquid biopsy via Guardant360 demonstrated the same Ex19del and T790M mutations as before. With no additional resistance mechanisms detected, we opted to rechallenge with osimertinib in order to simultaneously target both the sensitizing and resistance mutations. Osimertinib was re-introduced at 80 mg PO daily in conjunction with prednisone 40 mg PO daily on 24 July 2023. The prednisone was gradually tapered over 6 weeks and then stopped. This regimen was well tolerated with no laboratory evidence of hepatotoxicity or hepatitis and with minimal reported side effects. Follow-up imaging showed a remarkable response to the osimertinib rechallenge, including a significant improvement in both lung and leptomeningeal disease burden (Figure 1C,D).

However, after 8 months of osimertinib therapy, her disease began to progress, with worsening osseous metastasis noted on surveillance imaging in March 2024. Repeat NGS was performed again via liquid biopsy which revealed a newly developed EGFR C797S mutation. In response to this acquired osimertinib resistance, the patient was started on a chemotherapy regimen of carboplatin, pemetrexed, and bevacizumab in April 2024 in addition to continuing osimertinib, which was reduced to 40 mg PO daily for better tolerance. Surveillance imaging three months later in July 2024 showed largely stable disease with MRI of the brain actually demonstrating a decrease in her LMD burden. However, the patient tolerated this combination very poorly with severe emesis, anorexia, and weight loss, leading to discontinuation of chemotherapy at that time. She was switched to gefitinib plus osimertinib but despite aggressive supportive care her overall condition continued to deteriorate over the subsequent weeks and she ultimately opted for hospice comfort care in August of 2024.

## 3. Discussion

The initial NGS profiling of this patient’s lung adenocarcinoma identified concomitant Ex19del and T790M mutations. De novo compound mutations in the *EGFR* gene are fairly rare, with one recent meta-analysis of 46,679 EGFR mutation samples reporting that only 446 (~1%) harbored multiple de novo mutations [7]. Ex19del mutations are among the most common activating mutations in NSCLC and are generally associated with higher sensitivity to EGFR-TKIs [8]. In contrast, T790M mutations are well known for conferring resistance to first- and second-generation EGFR-TKIs, often emerging as an acquired mutation after treatment with these agents [9,10]. In view of the patient’s mutational profile, a third-generation EGFR-TKI such as osimertinib would have been an ideal first-line treatment. Osimertinib has demonstrated superiority over first-generation TKIs such as gefitinib and erlotinib in treatment-naïve advanced NSCLC [11] and has also proven more effective than chemotherapy with platinum-pemetrexed therapy in patients with disease progression after initial first-line EGFR-TKI treatment failure [12]. Osimertinib also possesses a unique pharmacologic profile that simultaneously targets both the activating Ex19del and resistance-promoting T790M mutations found in this patient, binding to T790M 17-fold tighter and 3-fold faster than WT EGFR [13]. Moreover, osimertinib has excellent CNS penetration and activity [14], an advantage that could have delayed or prevented the need for whole-brain radiation and its associated risks of cognitive dysfunction. Unfortunately, due to lack of insurance and coverage, this patient was not initially started on osimertinib and instead received it after multiple other treatments.

All drugs carry the risk of adverse events, though, and osimertinib-associated hepatitis, particularly in patients who have received prior immunotherapy, is an emerging topic of interest in the literature [6,15]. In one retrospective study, hepatotoxicity was significantly more frequent in patients who were directly transitioned from nivolumab to osimertinib (4/7 or 57.1%) as compared to those who received other additional agents between nivolumab and osimertinib treatment (2/40 or 5.0%) [14]. Another study found that 24% (5/21) of patients who received osimertinib within 3 months of PD-(L)1 blockade developed severe irAEs, but among those who received osimertinib first and then PD-(L)1 blockade, there were no severe irAEs (0/29) [6]. Interestingly, PD-(L)1 blockade followed by a different EGFR-TKI, such as afatinib or erlotinib, elicited no severe irAEs (0/27). Although the data is limited, it would appear that these adverse events are more common with osimertinib and are strongly associated with the initiation of osimertinib within a few months of IO treatment. Although the precise mechanisms underlying this association remain to be elucidated, the prolonged half-life of checkpoint inhibitors, which can take several months to fully wash out, is likely a significant contributor [16,17], and work is underway to identify which IO + TKI combinations are most likely to cause irAEs [18]. Unfortunately, a wash-out time of several months is often too long to wait in clinical practice. As more targeted cancer treatments become available, developing strategies to manage and work around specific adverse drug events will be essential in allowing us to always consider the best treatment for each patient’s unique presentation.

Notably, although there have been a few published case reports detailing successful osimertinib rechallenge following irAEs [19,20], our decision to re-initiate osimertinib at a full 80 mg dose, as opposed to slowly uptitrating as other groups have done, appears to be unique. Our report demonstrates the feasibility of this more aggressive rechallenging strategy, a potentially important factor in patients with significant time-sensitive disease burden. Furthermore, to our knowledge, this is the first report detailing the successful re-initiation of osimertinib in a patient with multiple de novo EGFR mutations and with significant leptomeningeal disease burden, adding a practical data point to the theoretical value of osimertinib in such a situation. As demonstrated by this patient’s treatment course, the decision to rechallenge with the best agent for a particular presentation, even if it means managing potentially serious side effects, can be pivotal. Six weeks into her initial treatment with osimertinib, the patient’s original care team had transitioned her to afatinib in order to avoid further hepatitis. However, the T790M mutation often confers resistance to second-generation TKIs [21] and although afatinib proved safe from a hepatitis standpoint, her cancer progressed with subsequent liver, bone, and leptomeningeal involvement. NSCLC with leptomeningeal metastases carries a particularly poor prognosis and remains notoriously difficult to treat once it develops [22,23]. That said, osimertinib has shown significant survival benefits and good tolerability for leptomeningeal NSCLC at both 160 mg daily [24] and 80 mg daily doses [25]. In light of this patient’s mutation profile and advanced metastases, the decision to restart osimertinib at a full 80 mg daily, monitoring for and managing potential adverse effects rather than opting for potentially less risky but also potentially less efficacious alternatives, gave our patient a remarkable 8-month period of improved disease burden and quality of life that she may not have otherwise had.

Unfortunately, this patient’s disease progressed following the emergence of a C797S mutation, which confers substantial resistance to osimertinib [26,27,28]. A combination of carboplatin, pemetrexed, and bevacizumab with 40 mg osimertinib daily stabilized the patient’s disease but was very poorly tolerated, eventually necessitating a non-chemotherapy alternative. Combining EGFR-TKIs from different generations has reportedly been successful in some similar cases [29], but the combination of gefitinib and osimertinib was ineffective in our patient, at least in part due to poor tolerance and compliance. Ultimately, this marked the end of our patient’s treatment course, highlighting the need for further research into overcoming osimertinib resistance. To date, amivantamab, with or without chemotherapy, has seen some success in this scenario (such as in the CHRYSALIS study [30]), but at present overcoming third-generation EGFR-TKI resistance remains a significant challenge in NSCLC treatment.

## 4. Conclusions

In summary, we have presented a case of osimertinib-induced hepatitis in a patient previously treated with immunotherapy that was successfully managed with osimertinib rechallenge in conjunction with a steroid taper. This approach warrants further investigation as the field attempts to better understand the frequency of osimertinib-related adverse events and the best strategies to mitigate and overcome them.

## Data Availability

Data pertaining to this report is available from the corresponding author upon reasonable request.
